# Age of onset of substance use and psychosocial problems among individuals with substance use disorders

**DOI:** 10.1186/s12888-016-1191-0

**Published:** 2017-01-11

**Authors:** Anju Poudel, Sital Gautam

**Affiliations:** Department of Nursing, Nepal Medical College, Jorpati, Kathmandu Nepal

**Keywords:** Age of initiation, Early onset, Late onset, Psychosocial problems, Substance use

## Abstract

**Background:**

Substance use is generally initiated in adolescence or early adulthood and is commonly associated with several physical, psychological, emotional and social problems. The objective of this study is to assess the age of onset of substance use differences on psychosocial problems among individuals with substance use disorders (SUDs) residing in drug rehabilitation centers.

**Methods:**

A descriptive cross sectional research design was carried out. Probability Proportional to Size (PPS) sampling technique was used to select the drug rehabilitation centers and all the respondents meeting the inclusion criteria of the selected seven rehabilitation centers were taken as a sample and comprised of 221 diagnosed individuals with SUDs. A semi structured self administered questionnaires were used to collect the information regarding demographic and substance use related characteristics. A standard tool Drug Use Screening Inventory-Revised (DUSI-R) was used to assess the psychosocial problems among individuals with SUDs. Data were analyzed using both descriptive and inferential statistics. Multivariate general linear model (MANOVA and MANCOVA) was used to evaluate differences in psychosocial problems between early vs late onset substance users.

**Result:**

The age of onset of substance use was significantly associated with psychosocial problems. The mean psychosocial problem scores were higher in early onset substance user (17 years or younger) than late onset substance user (18 years or higher) in various domains of DUSI-R even after controlling confounding factors. The two groups (early vs late) differed significantly in relation to age, gender, occupational status, current types of substance use, frequency of use, mode of substance use and relapse history.

**Conclusion:**

The study indicated that early onset substance users are at higher risk for psychosocial problems in various areas of life such as Behavior Pattern, Psychiatric disorder, Family system, Peer relationship, Leisure/Recreation and Work adjustment compared to late onset substance users. It highlights the need for early prevention, screening, and timely intervention among those individuals.

## Background

Substance use is associated with numerous undesirable short and long term consequences. There is considerable evidence that substance abuse among youth is widespread. Recent estimates indicate that the majority (74.0%) of substance abusers admitted in treatment center began substance use at the age of 17 or younger and 10.2% initiated use at the age of 11 or younger [1]. Study conducted among drug users in Nepal revealed that majority (95.0%) of the drug users initiated substance use before they reach 25 years. More than 81.2% drug users have experience of first time drug intake before they reach 20 years. More than 32% of drug users took drug first time in their life as early as 15 years [2].

Adolescence period is generally regarded as a critical risk period for the initiation of alcohol use, with multiple studies showing associations between age at first alcohol use and the occurrence of alcohol abuse or dependence [3, 4]. National Survey on Drug Use and Health data indicated that among those adults who first tried marijuana at the age of 14 or younger, 13.2% were classified with illicit drug dependence or abuse; this percentage was 6 times higher than that for adults who first used marijuana at the age of 18 or older [5].

Earlier onset of drug use is particularly predictive of long-term impairments [6, 7]. It is related to an elevated risk of substance use disorder (SUD) [8, 9], conduct disorder as well as school problems and risky sexual behaviors [10, 11]. Further, clients with early onset substance use are more likely to have family, social, and legal problems than their counterparts who initiate drug use later [10].

Adolescent illicit drug use is wide spread and has been associated with a variety of long term negative outcomes [12–14]. Previous studies showed associations between early initiation of illicit drug use and reduced educational and occupational attainment in adulthood [7, 12]. Early onset of drinking is also associated with a variety of other problematic outcomes later in adolescence and in young adulthood, including academic problems, dropping out of high school, delinquent behavior, substance use disorders, employment problems and unintentional injuries [8, 15]. The findings of a retrospective study, suggested that early drinking is strongly related with severity of substance use problems and reduced engagement in social or recreational activities, and weakly related with social and relational impairments including failure to fulfill obligations at work, school, or home and substance related involvement in legal problems [16].

A study on consequences of early onset cannabis use showed that individuals who started using cannabis prior to the age of 15 are at greater risk for later substance use, delinquency, truancy, and mental health problems including anxiety and depression [17]. Similarly, a longitudinal study demonstrated statistically significant bivariate associations between increasing levels of cannabis use at ages 14–21 and lower level of degree attainment and poor education by age 25; lower income at age 25; higher levels of welfare dependence; higher unemployment; lower levels of relationship satisfaction; and lower levels of life satisfaction [18]. This suggests that increasing cannabis use in adolescence and early adulthood is associated with a range of adverse outcomes in later life.

Contrast to all these findings, a study conducted in Quebac, Canada among students showed that early onset illicit drug use predicts conduct problems and school dropout, but not academic achievement and depressive symptoms [19]. However, most studies conducted in Nepal so far provide little or no information on these aspects. So, it is important to assess the consequences of early initiation of substance use at later life using the standard tool which compares the severity of problems among different domains of life among the individuals with substance use disorders in Nepal. The findings of the study might be beneficial to the policy makers, health care providers, psychologist and the team of the drug prevention and the rehabilitation centers to provide the holistic treatment approach to that group of individuals with SUDs who are more prone to have physical, social and psychological problems in life. Hence, the present study aims to

1. compare socio-demographic and substance use related characteristics between early and late onset substance users residing in treatment centers.
2. examine age of onset differences on psychosocial problem scores at various domains among treatment seeking individuals with SUDs.

## Methods

### Design

The study was conducted using descriptive cross sectional research design in August and September of 2015.

### Method

Among 22 drug rehabilitation centers operating in Kathmandu valley, five centers did not give permission to conduct the study. Therefore based on the feasibility of the study and number of clients residing in those centers, only seven centers were selected for the study using Probability Proportional to Size (PPS) sampling technique. Centers having few numbers of clients (less than 5) were not included in the study. Most of the centers provide residential rehabilitation care for an average of 3 months and all the centers had visiting psychiatrists, who on an absolute need-basis, sanctioned pharmacological treatment. Respondents who were willing to participate, free from any substance withdrawal delirium or ongoing psychotic symptoms but meet the criteria of substance use disorder were included in the study. Those admitted in treatment centers for more than 3 months were excluded to reduce recall bias. The total number of clients participated in final study were 221.

Data were collected by using Nepali version, pretested self administered questionnaire. Questions regarding socio-demographic characteristics and pattern of substance use were developed after extensive literature review. Regarding age of onset of substance use, there is no defined cut off age to indicate early and late onset. For this reason, early onset is defined as the one beginning at age 17 or younger and late onset as 18 or elder, which has been supported by the existing literatures [1, 5, 20, 21].

Socio-demographic characteristics included: Age; Gender (male, female); Educational level (≤secondary level, >secondary level); Occupation (Economically active, Economically inactive). Regarding education, respondents having education up to class 10 or below it were categorized as equal to or below secondary level and respondents having education above class 10 were categorized as above secondary level. Similarly, in reference to past 1 year status, students and unemployed were classified as economically inactive group whereas respondents engaged in some form of work were labeled as economically active group.

Substance use related characteristics included: Current types of substance use (licit, illicit, both licit and illicit); Current frequency of substance use (<3 times, ≥3 times); Duration of substance use; Relapse after first treatment (relapse, no relapse); Mode of substance use (injecting, non-injecting).

In this study substances were divided into licit (only alcohol), illicit (any illicit substances) and both licit and illicit substances. Tobacco use was not taken into consideration. Illicit substances comprises of opiates (opium, heroin, cough syrup, pain medications); stimulants (cocaine, crack, amphetamines and ecstasy); tranquilizers (diazepam, nitrazepam, alprazolam); inhalants (dendrite, paint thinner, varnish, petrol etc.); hallucinogens (LSD, acid, Ketamine) and cannabis.

Psychosocial problems of substance abusers were assessed by using a standard tool Drug Use Screening Inventory (DUSI-R), which was developed by Dr. Ralph Tarter in 1990. DUSI-R, a self-report questionnaire has dichotomous yes/no responses and quantifies severity of problems in 10 domains: 1) Substance Use, 2) Health Status, 3) Behavior Problems, 4) Psychiatric Disorder, 5) School Performance, 6) Family System, 7) Work Adjustment, 8) Peer Relationships, 9) Social Competence, and, 10) Leisure/recreation. **Substance use** – The substance use domain contains 15 items and measures the severity of substance use problems in relation to craving for substance use, withdrawal features, legal problems etc.; **Health Status** – The 10 items of this domain measures health status of individuals with SUDs; **Behavior pattern** – It contains 20 items that assess the characteristic pattern of behavior of the individuals with SUDs such as impulsivity, aggressiveness, emotional excitability etc.; **Psychiatric disorders** – This domain includes 20 items that measures the severity of emotional disturbances and psychiatric problems such as anxiety, depression, antisociality, psychotic symptoms etc.; **Social competence** – The 14 items of social competence measures the behavior of the members of society toward the individual’s personal and social skills required for adaptive functioning in society; **Family system** – The 14 items of family system assesses the organization of the family, behaviors of family members, pattern of communication, cohesiveness within the family and the relationship with the members; **School performance** – This domain is included only to those individuals who used to go to school within the past 1 year. Twenty items of school performance measures the severity of problems with respect to school environment, school engagement and academic performance; **Work adjustment** – Ten items of this domain are used only to those individuals who were engaged in some type of work or job within the past 1 year to measures the severity of problems related to work adjustment; **Peer relationship** – Fourteen items of this domain assesses the severity of problems in terms of peer selection, peer network and peer relation; **Leisure/Recreation** – Twelve items of this domain quantifies severity of disturbance in quality of activities during leisure time.

The scores on each domain and the overall problem density index ranged between 0 and 100%. The tool is copyright so permission for the use of the instrument was taken from the owner. Validity and reliability of the DUSI-R have been documented [22, 23]. For the use of the tool in Nepalese context, first forward translation (from English to Nepali) then backward translation (Nepali to English) was done. Finally, the back translated text was compared with the original text and differences between these two texts were resolved through discussion between translators for ensuring semantic equivalence. The back translated tool was also sent to the original developer of the tool for verification.

To identify the accuracy, adequacy and completeness of the tool, pre testing of the translated instrument was done on 15 respondents of one of the drug rehabilitation center. On the basis of pretesting, necessary modification were made in part I and II of the instrument. Cronbach’s alpha coefficient was computed to determine the reliability of translated (Nepali version) DUSI-R and was 0.89 which showed a high degree of internal consistency among the items. The alpha coefficient of different domain was as follows: Substance use = 0.79, Health status = 0.81, Behavior patterns = 0.82, Psychiatric disorder = 0.84, Social competence = 0.73, School performance = 0.81, Work adjustment = 0.78, Family system = 0.76, Peer relationship = 0.81, Leisure/Recreation = 0.74.

The school performance domain was used only by those respondents who used to go to school within the past 1 year. Similarly work adjustment domain was used by those respondents who were engaged in any form of work within the past 1 year. So, for the calculation of overall problem density index in those respondents who do not have to answer the question regarding that domain, the denominator was adjusted.

### Analysis

Data were analyzed by using in Statistical Package for Social Science (SPSS 20). Data were interpretative as higher the score higher the psychosocial problems. As the normality test of the data revealed normal distribution of the data, parametric tests were used for the analysis. For demographic and substance use related variables, independent sample t-tests were used to compare continuous variables and chi-square tests were used to evaluate categorical variables between groups. Multivariate general linear models (MANOVA, ANOVA, MANCOVA, ANCOVA) evaluated differences in psychosocial problem scores between early and late onset substance users. In all the inferential statistical procedures, *p* value of 0.05 or less (*p* ≤ 0.05) was considered statistically significant.

## Result

### Socio-demographic and substance use related characteristics

A total of 141 early onset substance users and 80 late onset substance users responded to the questionnaires making a response rate of 98.6%. In most of the characteristics the two groups differed significantly. The mean age was 25.77 ± 8.83 for early onset and 34.16 ± 8.72 for late onset substance users, which was statistically significantly. The early onset group consisted of higher percentage of male and economically inactive substance user compared to late onset group (Table 1).

**Table 1:** Characteristics of the respondents

Characteristics of respondents	Total n (%)	(Early onset) n (%)	(Late onset) n (%)	*P* value
Demographic characteristics				
Age of substance abusers in years	Mean ± SD	25.77 ± 8.83	34.16 ± 8.72	<0.001*
Gender (male)	190 (86%)	127 (90.1%)	63 (78.8%)	0.026*
Educational level				
> Secondary level	118 (53.4%)	74 (52.5%)	44 (55.0%)	0.780
Occupation				
Economically inactive	140 (63.3%)	101 (71.6%)	39 (48.8%)	0.001*
Substance use related characteristics				
Current type of substance use				
Licit	68 (30.8%)	21 (14.9%)	47 (58.8%)	
Illicit	56 (25.3%)	44 (31.2%)	12 (15.0%)	<0.001*
Both (Licit + Illicit)	97 (43.9%)	76 (53.9%)	21 (26.2%)	
Current frequency of substance use				
≥ 3 times	176 (79.6%)	128 (90.8%)	48 (60.0%)	<0.001*
Duration of substance use in years	(Mean ± SD)	8.43 ± 7.40	9.39 ± 6.99	0.348
Relapse after first treatment	100 (45.2%)	76 (53.9%)	24 (30.0%)	0.001*
Mode of substance use				
Injecting	57 (25.8%)	51 (36.2%)	6 (7.5%)	<0.001*

*n* = 221

*p significant at ≤ 0.05 level

The groups also differed significantly in relation to current types of substance use, frequency of substance use, mode of substance use and relapse history. A greater proportion of early onset substance users were both licit and illicit substance user, used most preferred substance more than or equal to three times per day, were injecting substance user and had relapse even after treatment. The group did not differ in educational level and duration of substance use (Table 1).

### Age of onset of substance use and psychosocial problem scores (DUSI-R domains scores)

MANOVA (multivariate analysis of variance) was performed to investigate age of onset of substance use differences in psychosocial problem scores. School performance and Work adjustment domains were not included in the analysis as the numbers of respondents in that domain were different than other domains. Therefore only eight domains: Substance use disorder, Behavior pattern, Psychiatric disorder, Health status, Family system, Social competence, Peer relationship and Leisure and recreation were used as dependent variables. The independent variable was age of onset of substance use: Early vs Late. Preliminary assumption testing was conducted to check for normality, linearity, univariate and multivariate outliers, homogeneity of variance-covariance matrices and multicollinearity, with no serious violations noted. There was a statistically significant differences between early onset and late onset substance users on the combined effects of eight domains of DUSI-R as dependent variables, (Wilks’ lambda = 0.738, *F* (8, 212) = 9.393, *p* < 0.001, partial eta squared = 262, power to detect the effect = 1.00).

Given the significance of the overall test, the univariate main effects were examined. The analysis revealed statistically significant differences between early and late onset substance users in all the domains of DUSI-R. Early onset users compared to late onset users displayed significantly (<0.05) higher scores on Substance use disorder, Behavior pattern, Psychiatric disorder, Health status, Social competence, Family system, Peer relationship and Leisure/recreation domains (Table 2).

**Table 2:** Age of onset of substance use (early vs late) differences on psychosocial problem scores (unadjusted for covariates)

Psychosocial problems (DUSI-R domains)	Early onset Mean ± SD	Late onset Mean ± SD	F	*P* value
Substance use disorder	80.42 ± 17.96	66.08 ± 23.81	25.550	0.000*
Behaviour pattern	72.51 ± 20.61	50.06 ± 25.07	42.843	0.000*
Psychiatric disorder	62.76 ± 20.99	47.31 ± 24.29	24.633	0.000*
Health status	59.78 ± 23.12	45.50 ± 27.09	17.172	0.000*
Social competence	65.60 ± 21.45	54.55 ± 23.07	12.812	0.000*
Family system	55.21 ± 22.61	37.14 ± 22.96	32.234	0.000*
Peer relationship	73.60 ± 20.25	50.44 ± 25.97	54.142	0.000*
Leisure/Recreations	74.29 ± 21.39	56.87 ± 23.26	31.732	0.000*
School performance	68.27 ± 23.02	50.95 ± 32.07	8.306	0.005*
Work adjustment	57.15 ± 26.77	31.91 ± 27.33	33.488	0.000*
Overall psychosocial problem	67.32 ± 16.34	49.57 ± 19.00	9.393	0.000*

*SD* standard deviation

*p significant at ≤ 0.05 level

A separate univariate analysis of variance (ANOVA) was also conducted, where School performance, Work adjustment and Overall psychosocial problem were dependent variable and age of onset of substance use (Early vs Late) was independent variable. The result indicated statistically significant differences in both the domains and overall psychosocial problems (Table 2).

### Age of initiation of substance use and psychosocial problem scores when controlling other variables

MANCOVA was employed to assess psychosocial problems among early and late onset substance user while controlling for other variables, such as age, gender, occupation, type of substance use, frequency of substance use, mode of substance use and relapse after treatment. The finding indicated statistically significant differences between early and late onset substance users on the combined effects of eight DUSI-R domains as dependent variables, (Wilks’ Lambda = 0.898; partial eta squared = 0.102; *F* (8204) = 2.910, *p* = 0.004. School performance and Work adjustment domain were not included in multivariate analysis as number of respondents in those domains differed from other domains. In follow up univariate analysis of covariance (ANCOVA) the two groups (early vs late) differed significantly on domains, Behavior pattern, Psychiatric disorder, Family system, Peer relationship and Leisure/Recreation. Early onset reported significantly higher problems than late onset substance users. However, age of onset differences were not noted in Substance use disorder, Health status and Social competence domains (Table 3).

**Table 3:** Age of onset of substance use (early vs late) differences on psychosocial problem scores controlling for other variables

Psychosocial problems (DUSI-R domains)	Early onset Mean ± SE	Late onset Mean ± SE	F	*P* value
Substance use disorder	77.08 ± 1.69	71.97 ± 2.38	2.560	0.111
Behaviour pattern	68.64 ± 1.84	58.88 ± 2.59	7.924	0.005*
Psychiatric disorder	59.67 ± 1.88	52.85 ± 2.64	3.721	0.050*
Health status	55.23 ± 2.05	53.52 ± 2.89	0.196	0.658
Social competence	62.60 ± 1.96	59.83 ± 2.76	0.562	0.454
Family system	53.91 ± 2.03	39.64 ± 2.85	13.785	0.000*
Peer relationship	67.93 ± 1.72	60.43 ± 2.41	5.387	0.021*
Leisure/Recreations	71.00 ± 1.91	62.66 ± 2.69	5.355	0.022*
School performance	67.40 ± 2.60	54.81 ± 6.38	2.950	0.089
Work adjustment	51.46 ± 2.80	39.28 ± 3.28	6.618	0.011*
Overall psychosocial problem	63.76 ± 1.39	55.85 ± 1.96	9.057	0.003*

*SE* standard error

*p significant at ≤ 0.05 level

Further analysis (ANCOVAs) was run in same manner for School performance, Work adjustment and Overall psychosocial problems with same covariates (age, gender, occupation, type of substance use, frequency of substance use, mode of substance use and relapse after treatment). The early onset differed significantly from late onset, on Work adjustment domain and Overall psychosocial problems. The differences in School performance domain remained insignificant while controlling covariates (Table 3).

Regarding covariates, the multivariate test result revealed statistically significant association between DUSI-R domains and covariates: types of substance use, frequency of substance use, gender, mode of substance use. Whereas occupation, age and relapse history of the respondents were not significantly associated. Significant covariates for each of the domain were: frequency and mode of substance use (Substance use domain); types of substance use and gender (Behavior pattern domain); types of substance use, mode of substance use and gender (Psychiatric domain, Health status domain, Overall psychosocial problem); types of substance use and mode of substance use (Peer relationship and Work adjustment domains); frequency of substance use and gender (Leisure/Recreation domain); gender only (School performance domain).

## Discussion

The result indicated differences in the demographic and substance use related characteristics between early and late onset substance users. The characteristics differed significantly in relation to age, gender, occupation, current types of substance use, frequency of substance use, mode of substance use and relapse history. Higher percentage of early onset substance user were younger, male, economically inactive, both licit and illicit substance user, injecting drug user, used most preferred substances more than three times per day, and had the history of relapse event after first treatment. This signifies that when individuals initiate substance use early in life, it is associated with more problematic form of substance use [1, 8, 9, 12, 16].

The age of onset of substance use was significantly associated with psychosocial problems. The respondents who self reported initiating substance use before 18 years had significantly higher problems on overall and all the domains of DUSI-R such as Substance use disorder, Behavior pattern, Psychiatric disorder, Health status, Social competence, Family system, Peer relationship, Leisure/Recreation, School performance and Work adjustment domains. When demographic and substance use related characteristics such as age, gender, occupation, types of substance use, frequency of substance use, mode of substance use and relapse history were entered as covariates, the overall model still suggested differences in psychosocial problems among early and late onset of substance user. However, in the follow up ANCOVA, previously observed statistically significant group differences in psychosocial problem scores (DUSI-R domains), were not observed in some domains such as Substance use disorders, Health status, Social competence and School performance. The group differences in those domains were apparently not due to the effects of age of onset of substance use per se, but may be attributed to the effects of the eight covariates. Whereas, differences in overall psychosocial problem score and different domains such as Behavior pattern, Psychiatric disorder, Family system, Peer relationship, Work adjustment and Leisure/recreations were still observed even after controlling the effects of covariates.

Finally, in agreement with previous studies, the present study indicated that early onset substance user had significantly higher psychosocial problem scores in domains such as Behavior Pattern, Psychiatric disorder, Family system, Peer relationship, Work adjustment and Leisure/Recreation compared to late onset substance user [16–19, 24]. The findings of this study is also supported by other studies which showed that early onset substance users have a higher risk of behavioral problems such as impaired executive function and impulse control problems [24], incarceration due to crime [12, 15, 25], and conduct disorder [10, 11, 19]. Study from Virginia also showed that early drinking was associated with reduced engagement in social activities [16]. Although most of the studies [3, 4, 26] showed significant difference between early and late onset substance user in problems related to substance use disorder, the present study did not show any difference in that domain when other independent variables were controlled. It might be due to the effects of other variables in some studies as well as difference in sample size and tools used to assess psychosocial problems.

### Limitations

The findings of this study should be interpreted in light of their limitations. Due to the design of this study, causality cannot be determined. Regarding frequency of substance use, the present study is based on number of times of substance use per day without much regard to quantity of substance use. This could have influenced the information regarding this variable. Few number of female substance user might have limited the significance of differences found in gender which could have influenced further findings. The findings relies on respondents’ self-reports, which may be influenced by under-reporting and memory errors, although it was minimized by excluding the individuals who were admitted in rehabilitation centers for more than 3 months.

## Conclusion and implication

The findings of this study have supported the results of numerous cross-sectional and longitudinal studies that age of onset of substance use is significantly associated with psychosocial problems. The early onset substance users have more problematic substance use behaviors and are at risk for more psychosocial problems at various areas life such as behavior pattern, psychiatric disorder, family system, peer relationship, work adjustment and leisure/recreation. The study suggests that prevention and educational efforts must be initiated in early adolescence and should be targeted towards preventing initiation of the use of licit and illicit substances that are legal and commonly used by adolescence. Since early age of onset of substance use is associated with the future risk, intensity, complexity substance use disorders and related life problems, it highlights the need for early prevention, screening, and timely intervention among those individuals. This group of substance users also warrants additional clinical assessments and interventions to facilitate treatment engagement and deliver coordinated care for co-morbid conditions.

## Abbreviations

ANCOVA: Analysis of covariance
ANOVA: Analysis of variance
DUSI-R: Drug Use Screening Inventory-Revised
MANCOVA: Multivariate analysis of covariance
MANOVA: Multivariate analysis of variance
PPS: Probability Proportional to Size
SD: Standard deviation
SE: Standard error
SPSS: Statistical Package for Social Sciences
SUDs: Substance use disorders
